# Needle and Syringe Preference in Cosmetic Botulinum Toxin Treatment of the Upper Face: A Randomized, Controlled, Single‐Blind Study

**DOI:** 10.1111/jocd.71052

**Published:** 2026-07-17

**Authors:** Goknil Gultekin, Alper Koycu, Serhat İnan, Adnan Fuat Buyuklu

**Affiliations:** ^1^ Private Otorhinolaryngology Practice İstanbul Turkey; ^2^ Department of Otolaryngology, Head and Neck Surgery Baskent University Faculty of Medicine Ankara Turkey; ^3^ Private Otorhinolaryngology Practice Ankara Turkey

**Keywords:** Botox, bruising, needle, pain, upper face

## Abstract

**Background:**

The tools selected for botulinum toxin application can affect patient satisfaction and financial efficiency due to injector dead space, especially with high‐priced medications.

**Objective:**

To compare two injection systems used for upper‐face botulinum toxin applications—a 34‐gauge needle combined with a low dead space injector (LDSI) and a 30‐gauge fixed‐needle insulin syringe—with respect to pain, ecchymosis, and product wastage.

**Methods:**

This prospective, randomized, controlled, single‐blind clinical study involved 42 patients. The upper facial region of each patient was divided into symmetric halves. On the study side, botulinum toxin was administered using a 34‐gauge needle connected to an LDSI; on the control side, a 30‐gauge fixed‐needle insulin syringe was used. Pain, bruising, and product wastage were compared between the two sides.

**Results:**

The pain scores were lower across all three regions on the study side than in the control side. Bruising scores were lower on the periorbital and frontal regions on the study side. The control side demonstrated an approximately 2.8‐fold higher product wastage than that of the study side.

**Conclusion:**

The use of a 34‐gauge needle combined with an LDSI for upper‐face botulinum toxin injections may improve patient comfort by reducing pain and bruising, while also decreasing product wastage. These findings should be interpreted as reflecting the performance of the injection system as a whole rather than needle diameter alone.

## 1 Introduction

Botulinum toxin application in facial plastic surgery is one of the most common non‐surgical rejuvenation procedures globally. Administration through fine needles is recommended to prevent vessel injury and ecchymosis [1, 2].

Most clinicians prefer using single‐use insulin or tuberculin syringes with 30‐gauge (G) needles during administration of botulinum toxin to the face. However, some clinicians have reported that patients experience less pain.

Another important consideration is product or dose conservation. Every syringe holds a space between the needle and the plunger that prevents the complete delivery of the medication to the target tissue. This dead space accounts for residual product left in each syringe. Syringes with low dead volume, or low dead space injectors (LDSIs) have been produced and are recommended to provide precise dosing and minimize product wastage [3, 4, 5, 6, 7, 8, 9, 10, 11].

Herein, we investigated the differences between two injection systems used in cosmetic upper‐face neurotoxin application: a 34‐gauge needle combined with an LDSI and a 30‐gauge fixed‐needle insulin syringe, in terms of pain, ecchymosis, and product wastage. To our knowledge, no prospective studies have reported on these parameters in a single‐blind, randomized group. We hypothesized that using a 34‐gauge needle and an LDSI in upper‐face neurotoxin application would result in significantly less pain, fewer vascular traumatic events such as bruising or hematoma, and a significant reduction in product wastage due to dead space.

Botulinum toxin injections are influenced by multiple technical and solution‐related factors beyond needle diameter alone. Pain perception, in particular, may vary depending on parameters such as solution pH, dilution status, injection speed, and individual patient sensitivity. Therefore, evaluating injection systems in a controlled clinical setting is essential to better understand the combined effect of device‐related variables on patient experience and treatment efficiency.

## 2 Materials and Methods

### 2.1 Study Population and Ethical Approval

The study was conducted among healthy individuals aged 18–65 years who presented to our otolaryngology clinic for upper‐face cosmetic botulinum toxin application in Baskent University Ankara Hospital, Turkey.

The prospective, randomized controlled, single‐blind clinical study was approved by the Baskent University Clinical Research Ethics Committee and the Turkish Medicines and Medical Devices Agency (project number: E‐61749811‐000‐1 056 838) and supported by the Baskent University Clinical Research Fund. The study was conducted in accordance with the principles of the Declaration of Helsinki. Detailed informed consent was obtained from each participant, and the procedural steps were explained to the patients by the primary investigator.

### 2.2 Study Design

**FIGURE 1:**
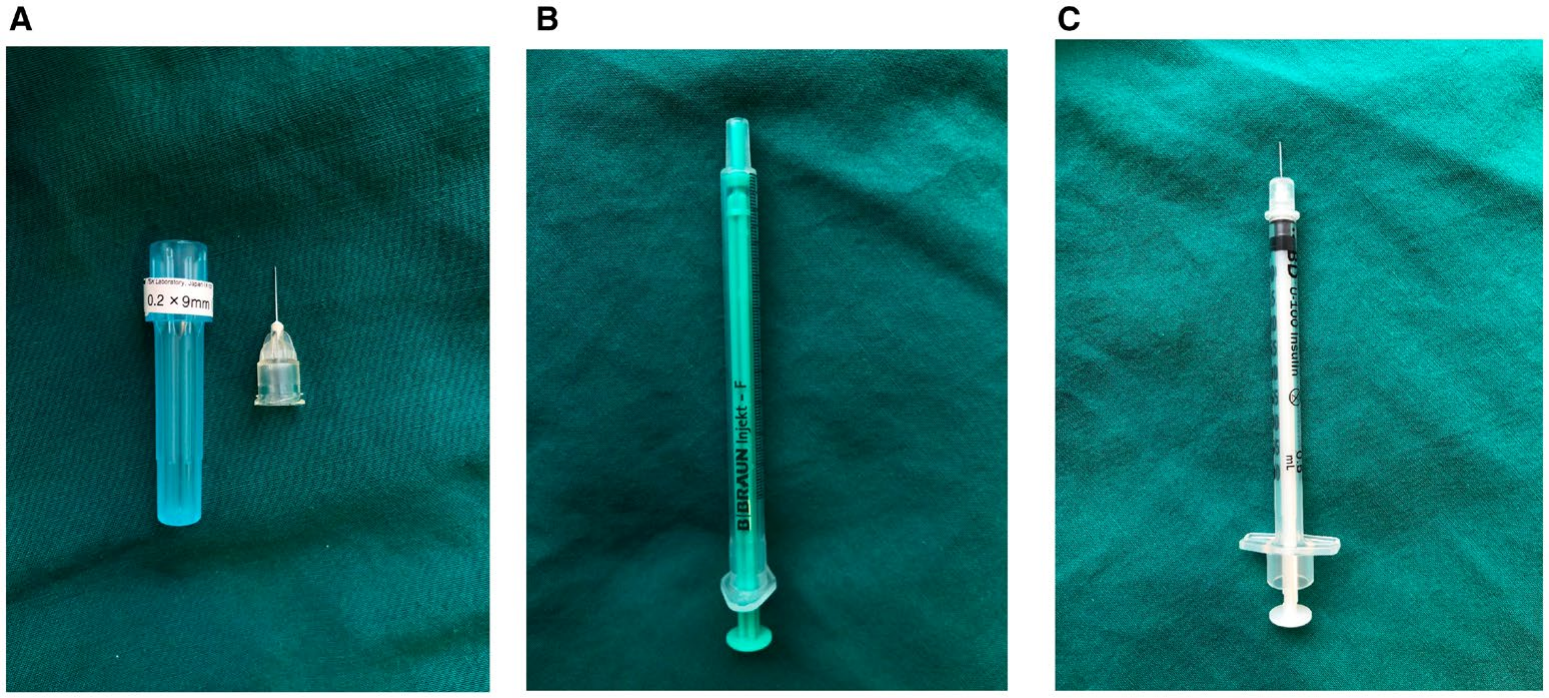
(A) 34‐gauge needle (The Invisible needle, TSK Laboratory‐Japan). (B) A low dead space injector (Injekt‐F Luer Solo, B. Braun, 1 mL). (C) A 30‐gauge fixed‐needle insulin syringe (BD Ultrafine 8 mm × 30‐gauge).

The inclusion and exclusion criteria are shown on Table 1. Cosmetic botulinum toxin application was performed on the upper facial region of all participants. The upper facial region of the participants was divided into two symmetric halves, right and left facial halves, and the application for the study side was carried out using a 34‐gauge needle (The Invisible needle, TSK Laboratory‐Japan) connected with a low dead space injector (LDSI) (Injekt‐F Luer Solo, B. Braun, 1 mL). For the control side, a fixed‐needle insulin syringe (BD Ultrafine 8 mm × 30‐gauge) was used. In this system, the needle is permanently attached to the syringe and cannot be detached or reconnected. Following complete removal of the vial stopper, the botulinum toxin solution was directly aspirated into the syringe without intermediate transfer steps (Figure 1A–C). Patients were randomized into two groups (group 1 and group 2) using a computer‐generated program. The right facial half of group 1 patients and the left facial half of group 2 patients were designated as the study side, while the contralateral facial halves were designated as the control side. The patients were blinded to the group allocations of the facial halves.

**TABLE 1:** Inclusion and exclusion criteria.

Inclusion criteria	Exclusion criteria
18–65 years old healthy volunteers (patients)	Patients who has botulinum toxin application to their face on the last 6 months
Accepting the cosmetic botulinum toxin application to the upper face and	Patients who has ablative laser treatment, radiofrequency treatment, chemical peeling to their face on the last 6 months
	Patients who has temporary or semi dissolving cosmetic filler applications to their face on the last 1 year
	Patients who has non dissolvable filler applications to their face in the past
	Patients who suffer from a rheumatological disease (Skleroderma, Rheumatoid Artritis etc.)
	Patients who has a contraindication to neurotoxin application
	Patients who has bleeding disorder or using any medication may cause bleeding problem
	Patients who has on their menstruation bleeding period

This study compares two injection systems rather than an isolated variable: a 34‐gauge needle combined with a low dead space injector (LDSI) and a 30‐gauge fixed‐needle insulin syringe.

### 2.3 Botulinum Toxin Injection Protocol

Botulinum toxin injections were administered to the target muscles in the frontal, glabellar, and periorbital regions. For the initial treatment, the target muscles and treatment scheme were kept the same for all patients, and the same unit‐based toxin doses were used according to consensus papers and guidelines [1, 2, 12, 13]. One week later, patients were called for follow‐up, additional doses and additional points were provided if needed. The botulinum toxin used in the study (100 U Botox‐Allergan) was diluted with 2.5 mL of physiologic saline (0.9% NaCl solution) according to the standard dilution protocol. The plastic‐sponge cap of the toxin vial was completely removed, and the syringes were filled directly from the vial, thus preventing possible blunting of the needle tips before coming into contact with the patient. For each patient, six injectors were prepared: three for the study side and three for the control side, to be used in the frontal, glabellar, and periorbital areas. Each syringe and its attached needle were used only once for one specific target region, and refilling from the toxin vial into the same injector was not permitted. Care was taken to ensure no air bubbles in the injectors during filling, and the entire content of the injector was used for each target area.

**FIGURE 2:**
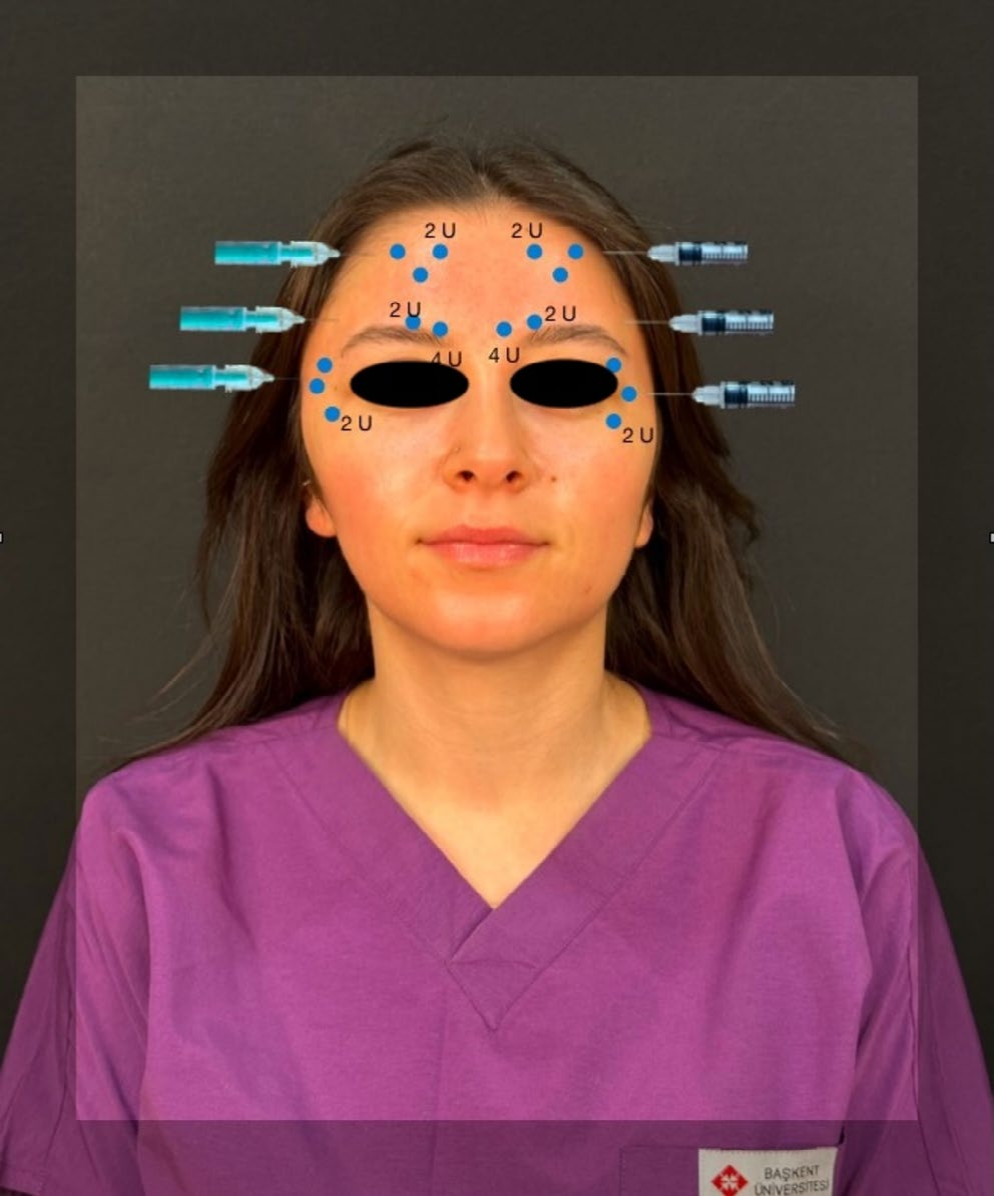
Treatment plan for a patient whose right facial half was designated as the study side and left facial half as the control side.

A detailed scheme for the treatment was explained on a model (Figure 2). All injections were administered by a right‐hand dominant investigator (GG) in an upright position on the same examination chair. Additional pain control measures such as ice, vibration, or topical anesthesia were not applied before or after the procedure. Each patient initially received treatment on the right half of the face, followed by treatment on the left half, ensuring that 21 patients received treatment on the study side first and 21 received treatment on the control side first to avoid the bias. The application sequence was consistent for each patient, starting with the glabellar area, followed by the frontal and periorbital areas.

### 2.4 Follow‐Up, Pain Assessment and Post‐Botulinum Bruising

A visual analog scale (VAS) was used for pain assessment, with scores ranging from 0 (no pain) to 10 (severe pain). At the end of the procedure, patients were instructed to compare and rate the pain they felt in the frontal, glabellar, and crow's feet regions on the right and left halves of their faces.

Bruising and hematoma were evaluated and scored during the follow‐up visit on the first day after the procedure, using the following scoring system: 0 (no bruising), grade 1 (mild bruising, < 5 mm diameter), grade 2 (noticeable bruising, 5–10 mm diameter), grade 3 (hematoma, > 10 mm diameter).

### 2.5 Assessment of Residual Product in the Dead Space

**FIGURE 3:**
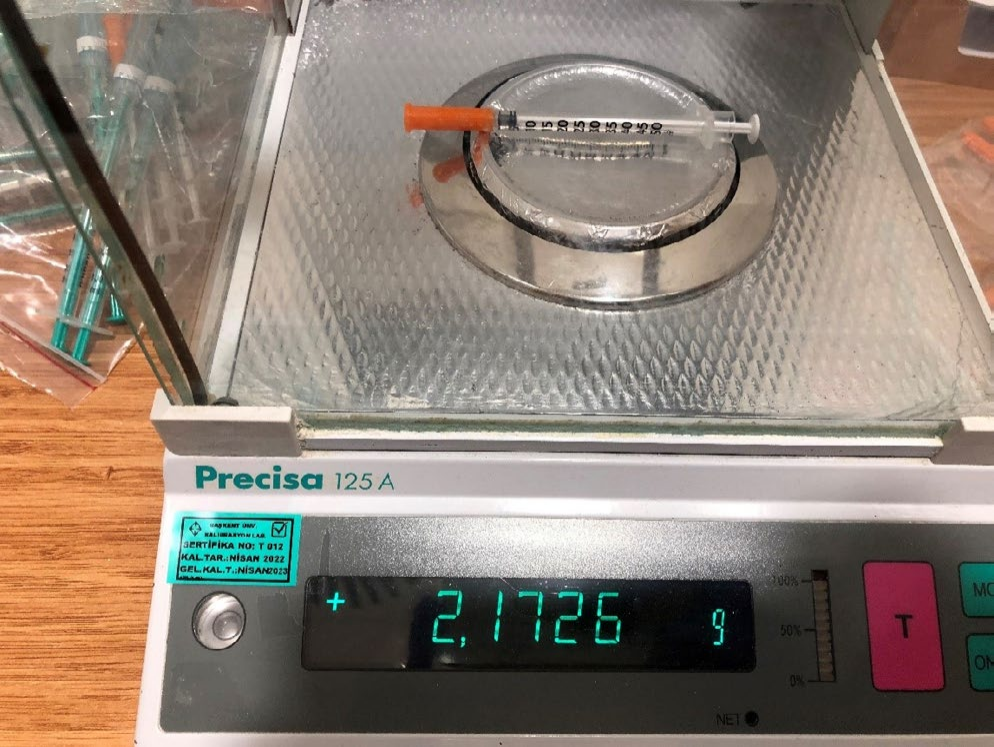
Measurement of the weight of the 30‐gauge syringe containing the remaining solution using a microbalance scale.

To analyze cost‐effectiveness, measurements were performed using a microbalance scale (Precisa 125A SCS Digital Analytical Balance Scale). The used injectors from the study and control sides were labeled according to the treated region and stored in airtight sealed bags. In addition, a new unused injector of each type was weighed as a reference. Subsequently, the injectors used on the right and left halves of the patient's face, which were assumed to have delivered the full injectable product, were individually weighed to determine the amount of residual product in the dead space (Figure 3).

### 2.6 Statistical Analysis

All statistical analyses were performed using SPSS version 22.0 (SPSS Inc., Chicago, IL, USA). Descriptive data were presented as mean ± standard deviation (SD). The chi‐square test was used to evaluate associations between categorical variables. Non‐normally distributed paired data were analyzed using the Wilcoxon signed‐rank test. A *p* value of < 0.05 was considered statistically significant.

## 3 Results

Volunteers were recruited for upper facial botulinum toxin application at the Department of Otolaryngology, Baskent University, between June 2022 and August 2022. According to the research hypothesis, with Cohen's d effect size of 0.80, a type I error of 0.05, and test power of (1‐𝜷) = 0.80, this study included 42 patients, comprising 11 male and 31 female patients, with mean ages of 39.5 and 41.9 years, respectively.

The study examined differences in pain scores, bruising, and the amount of residual botulinum toxin referred to as lost dose between the two halves of the patients' faces, which were treated with different instruments. When examining the frontal region in terms of pain, the study side (Frontal i) had pain scores (VAS) ranging from 1 to 7, with a mean score of 3.90 (SD ±1.52). Conversely, the control side (Frontal c) had scores ranging from 2 to 8, with a mean score of 5.66 (SD ±1.58).

For the glabellar region, the study side (Glabellar i) had VAS scores ranging from 0 to 8, with a mean score of 3.30 (SD ±1.50). The control side (Glabellar c) had VAS scores ranging from 1 to 8, with a mean score of 5.07 (SD ±1.88).

For the periorbital region, the study side (Periorbital i) had VAS scores ranging from 1 to 5, with a mean score of 2.0 (SD ±1.22) across the 42 half‐faces. The control side (Periorbital c) had VAS scores ranging from 1 to 8, with a mean score of 3.33 (SD ±1.95).

In all upper facial regions considered in the study, the pain scores on the study side were significantly lower than those on the control side (*p* < 0.05) (Table 2).

**TABLE 2:** Statistical difference between study and control sides.

	N (number of study and control sides)	Mean VAS	*p* (VAS)	Mean eccymosis score	*p* (eccymosis)	Remaining product in the dead space	*p* (Remaining product)
Frontal i	42	3.90 ± 1.52	*p* < 0.05	0	*p* < 0.05	0.0189 ± 0.0124	*p* < 0.05
Frontal c	42	5.66 ± 1.58		0.14		0.0501 ± 0.0460	
Glabellar i	42	3.30 ± 1.50	*p* < 0.05	0	*p* > 0.05	0.0182 ± 0.0124	*p* < 0.05
Glabellar c	42	5.07 ± 1.88		0.04		0.0578 ± 0.0505	
Periorbital i	42	2.0 ± 1.22	*p* < 0.05	0	*p* < 0.05	0.0207 ± 0.0167	*p* < 0.05
Periorbital c	42	3.33 ± 1.95		0.33		0.0539 ± 0.0505	

*Note:* Values are presented as mean ± standard deviation (SD). *p* values were calculated using the Wilcoxon signed‐rank test or the chi‐square test, as appropriate. i: study side; c: control side.

Abbreviation: VAS, Visual analog scale.

When examining ecchymosis, the study side (Frontal i) in the frontal region had no bruising (grade 0). Grade 1 ecchymosis was observed in six patients on the control side (Frontal c) of the frontal region.

For the glabellar region in terms of ecchymosis, no ecchymosis was observed on the study side (Glabellar i), while on the control side (Glabellar c), grade 1 ecchymosis was observed in two patients.

In the periorbital region, no ecchymosis was observed on the study side (Periorbital i), while on the control side (Periorbital c), grade 1 ecchymosis was observed in eight patients and grade 2 ecchymosis was observed in three. The study and control sides on the frontal region (*p* = 0.014) and periorbital region (*p* = 0.02) differed significantly in terms of ecchymosis but not on the glabellar region (*p* = 0.157) (Table 2).

In terms of dead space volume, the remaining product in the needle and syringe was evaluated after injection of the entire injectable product. For the frontal region, the average remaining product in the syringe was 0.0189 g (SD ±0.0124) for the study side and 0.0501 g (SD ±0.0460) for the control side (*p* = 0.02). For the glabellar region, the average remaining product in the syringe was 0.0182 g (SD ±0.0124) for the study side and 0.0578 g (SD ±0.0559) for the control side (*p* < 0.01). For the periorbital region, the average remaining product in the syringe was 0.0207 g (SD ±0.0167) for the study side and 0.0539 g (SD ±0.0505) for the control side (*p* = 0.01). The amount of remaining product in the syringe differed significantly between the study and control sides (*p* < 0.05) (Table 2).

## 4 Discussion

In this study, in upper‐face neurotoxin applications, lower VAS pain scores were observed across all regions (frontal, glabellar, periorbital) when injections were performed using a 34‐gauge needle combined with a low dead space injector compared to a 30‐gauge fixed‐needle insulin syringe, and this difference was statistically significant for all assessed upper facial regions. The incidence of ecchymosis differed significantly in the frontal and periorbital regions, whereas no statistically significant difference was observed in the glabellar region.

Although it may appear intuitive that smaller‐diameter needles are associated with reduced pain perception, our findings provide controlled clinical evidence supporting this relationship within a prospective, randomized, split‐face design. This design minimizes interpatient variability and strengthens the reliability of the comparison. However, it is important to emphasize that pain perception in injectable treatments is multifactorial and may also be influenced by factors such as solution pH, dilution status, injection speed, depth, and individual patient sensitivity.

These findings are consistent with previous studies. In 2014, Sezgin et al. examined the effect of needle thickness on pain and bruising in minimally invasive facial procedures and reported lower pain scores with finer needles, although statistical significance was limited to the frontal region [14]. Similarly, Alam et al. demonstrated significantly lower pain scores with a 32‐gauge needle compared to a 30‐gauge needle in neurotoxin injections [15]. Regarding ecchymosis, prior studies have generally reported no significant differences between needle sizes [14, 16]. In our study, ecchymosis was significantly lower in the frontal and periorbital regions, while lower rates were observed in the glabellar region without reaching statistical significance. Although ecchymosis is typically mild and self‐limiting in botulinum toxin applications, it remains a relevant concern for patients in aesthetic practice.

In our study, due to dead space volume, an average of 0.0539 g of product loss was observed with a 30‐gauge fixed‐needle insulin syringe across all regions, whereas an average of 0.0192 g of product loss was observed when using a low dead space injector combined with a 34‐gauge needle. Accordingly, the amount of wasted product was approximately 2.8 times higher with the standard syringe system. Although this finding is expected given the structural characteristics of low dead space injectors, our study provides a quantitative clinical assessment of this difference in the context of aesthetic botulinum toxin applications.

Using a microbalance scale, we assessed 100 units of botulinum toxin diluted with 2.5 mL of saline and determined that 2 units of Botox corresponded to a weight of 0.0790 g. Based on this calculation, the average remaining solution of 0.0539 g in the control syringe was equivalent to approximately 1.36 units of botulinum toxin, whereas the average remaining solution of 0.0192 g in the low dead space injector corresponded to approximately 0.48 units. Based on approximate contemporary market pricing, the product loss due to dead space translated into an estimated cost of approximately 1.36 USD per syringe in the control group and 0.48 USD in the study group. These values are intended to provide a practical estimation rather than an exact economic calculation. To our knowledge, few studies have quantified the financial implications of dead space in aesthetic botulinum toxin practice, and this aspect represents one of the clinically relevant contributions of our study.

Importantly, the findings of this study reflect the combined effect of needle gauge and syringe design rather than an isolated variable. In particular, the reduction in product wastage is most likely attributable to the low dead space characteristics of the injector rather than needle diameter alone. Therefore, the observed advantages should be interpreted as properties of the injection system as a whole. Previous studies have typically evaluated needle diameter using a constant syringe system, whereas our study reflects real‐world clinical practice in which needle and syringe are used together as a combined device. Recent studies have also highlighted the importance of device‐related variables in botulinum toxin applications [17, 18, 19, 20, 21, 22].

Nevertheless, several limitations of this study should be acknowledged. First, the study evaluates a combined needle–syringe system rather than isolating needle diameter as a single variable; therefore, the observed differences, particularly in product wastage, may be influenced by the injector design rather than needle gauge alone. Second, pain assessment was based on subjective VAS scores, and variability in individual pain thresholds may have affected the results. In addition, although measurements of residual product were performed using a microbalance scale under controlled conditions, minimal evaporation or handling‐related variations cannot be completely excluded. Future studies involving larger and more homogeneous patient populations, as well as designs isolating individual device components, would be valuable to further clarify these findings.

## 5 Conclusions

In upper‐face botulinum toxin applications, the use of a 34‐gauge needle combined with a low dead space injector was associated with lower pain scores across the frontal, glabellar, and periorbital regions compared with a 30‐gauge fixed‐needle insulin syringe. Ecchymosis was significantly lower in the frontal and periorbital regions, whereas the difference in the glabellar region was not statistically significant. In addition, the 34‐gauge needle–LDSI combination resulted in less product wastage and may provide more efficient toxin utilization. These findings reflect the performance of the combined injection system rather than needle diameter alone.

## Author Contributions

Goknil Gultekin wrote the original draft, collected the data and performed the analysis. Adnan Fuat Buyuklu conceptualized the study. Alper Koycu, Serhat İnan supervised the research and critically revised the manuscript. All authors reviewed and approved the final version.

## Funding

This study was supported by Baskent University Research Fund with project number K (A 21/179).

## Ethics Statement

Ethics approval was obtained from Baskent University Clinical Research Ethics Committee (project number: KA 21/179) and the Turkish Medicines and Medical Devices Agency with approval number E‐61749811‐000‐1 056 838.

## Consent

Informed consent has been obtained from all individuals included in this study. Written informed consent was obtained from the patient whose photograph was used in Figure 2 for publication of the image in this article.

## Conflicts of Interest

The authors declare no conflicts of interest.

## Data Availability

The data that support the findings of this study are available from the corresponding author upon reasonable request.
